# Probiotic Potential and Safety Evaluation of *Enterococcus faecalis* OB14 and OB15, Isolated From Traditional Tunisian Testouri Cheese and Rigouta, Using Physiological and Genomic Analysis

**DOI:** 10.3389/fmicb.2019.00881

**Published:** 2019-04-24

**Authors:** Olfa Baccouri, Amine Mohamed Boukerb, Leila Ben Farhat, Arthur Zébré, Kurt Zimmermann, Eugen Domann, Mélyssa Cambronel, Magalie Barreau, Olivier Maillot, Isabelle Rincé, Cécile Muller, Mohamed Nejib Marzouki, Marc Feuilloley, Ferid Abidi, Nathalie Connil

**Affiliations:** ^1^Laboratory of Protein Engineering and Bioactive Molecules (LIP-MB), National Institute of Applied Sciences and Technology, University of Carthage, Tunis, Tunisia; ^2^Laboratoire de Microbiologie Signaux et Microenvironnement (LMSM) EA 4312, Normandie Université – Université de Rouen, Évreux, France; ^3^SymbioPharm GmbH, Herborn, Germany; ^4^Institute of Medical Microbiology, German Centre for Infection Research, Justus-Liebig-University Giessen, Giessen, Germany; ^5^UNICAEN, U2RM, Normandie Université, Caen, France

**Keywords:** Testouri cheese, Rigouta, *Enterococcus faecalis*, probiotics, antibiotic resistance, genomic analysis, virulence, safety

## Abstract

Lactic acid bacteria (LAB) strains OB14 and OB15 were isolated from traditional Tunisian fermented dairy products, Testouri cheese and Rigouta, respectively. They were identified as *Enterococcus faecalis* by the MALDI TOF-MS (matrix assisted laser desorption-ionization time of flight mass spectrometry) biotyper system and molecular assays (species-specific PCR). These new isolates were evaluated for probiotic properties, compared to *E. faecalis* Symbioflor 1 clone DSM 16431, as reference. The bacteria were found to be tolerant to the harsh conditions of the gastrointestinal tract (acidity and bile salt). They were low to moderate biofilm producers, can adhere to Caco-2/TC7 intestinal cells and strengthen the intestinal barrier through the increase of the transepithelial electrical resistance (TER). Susceptibility to ampicillin, vancomycin, gentamicin and erythromycin has been tested using the broth microdilutions method. The results demonstrated that *E. faecalis* OB14 and OB15 were sensitive to the clinically important ampicillin (MIC = 1 μg/mL) and vancomycin (MIC = 2 μg/mL) antibiotics. However, Whole Genome Sequencing (WGS) showed the presence of tetracycline resistance and cytolysin genes in *E. faecalis* OB14, and this led to high mortality of *Galleria Mellonella* larvae in the virulence test. Hierarchical cluster analysis by MALDI TOF-MS biotyper showed that *E. faecalis* OB15 was closely related to the *E. faecalis* Symbioflor 1 probiotic strain than to OB14, and this has been confirmed by WGS using the average nucleotide identity (ANI) and Genome-to-Genome Hybridization similarity methods. According to these results, *E. faecalis* OB15 seems to be reliable for future development as probiotic, in food or feed industry.

## Introduction

Enterococci are normal inhabitants of the gastrointestinal tract of humans and animals. These bacteria are ubiquitous in nature and have been used in the food industry as probiotics (Franz et al., 2003), or as starter culture in manufacturing cheese in high salt content and low pH (Wessels et al., 1991). They contribute to the characteristic taste of traditional Mediterranean cheeses and can be present in other fermented foods such as sausages, olives, and vegetables. Some strains have been proposed for food preservation, as they produce bacteriocins (enterocins), antimicrobial peptides with the potential to inhibit the growth of food-borne pathogenic and spoilage bacteria (Khan et al., 2010; Franz et al., 2011). Recently, bacteriocins have been suggested as a new probiotic trait when selecting beneficial microbes. Most of the enterocins belong to Class-II bacteriocins, including the pediocin family of enterocins highly active against *Listeria* spp (Ness et al., 2014). Some enterococci strains produce simultaneously several bacteriocins, which give them a competitive advantage toward other microbes for colonization and niche control (Hanchi et al., 2018).

While the genus *Enterococcus* includes many species, only few have been studied to be used as probiotics, such as *E. faecalis*, *E. faecium*, *E. lactis* and more recently *E. hirae* (Adnan et al., 2017) and *E. durans* (Li et al., 2018). For example, some *Enterococcus* strains are currently in use as therapeutic treatments, marketed as Cylactins (Hoffmann-La Roche, Basel, Switzerland), Fargo 688s (Quest International, Naarden, Netherlands), ECOFLOR (Walthers Health Care, DenHaag, Netherlands), or Symbioflor 1 (SymbioPharm, Herborn, Germany), to alleviate the symptoms of irritable bowel syndrome and recurrent chronic sinusitis or bronchitis (Franz et al., 2003; Foulquié Moreno et al., 2006). The probiotic *E. faecalis* Symbioflor 1 clone DSM 16431, originally isolated in the 1950s from the stool sample of a healthy human adult, has been used as a probiotic for more than 50 years without any report or documentation of any infection or adverse effect. The complete genome sequence of this strain has been determined and several toxicological studies showed that the strain can be administred safely to humans (Domann et al., 2007; Christoffersen et al., 2012; Fritzenwanker et al., 2013). However, according to FDA, probiotic shall be used carefully by populations at risk, for example those with immune compromise, premature infants, patients suffering from various pathologies…

On the other hand, some enterococci are known to be opportunistic pathogens and are a prevalent cause of nosocomial infections. These bacteria have been implicated in bacteraemia, endocarditis, urinary tract infections or other infections. Recently, a role of *E. faecalis* in pancreatic and colorectal cancers has also been suggested but this remains controversial (De Almeida et al., 2018; Maekawa et al., 2018). One factor which contributes to the pathogenicity of *E. faecalis*, is the resistance of many strains to a broad range of antibiotics (Franz et al., 2003). This antibiotic resistance is due to intrinsic and/or acquired genes located on the chromosome, or on mobile elements including plasmids and transposons. Vancomycin-resistant enterococci pose major problems in treating human clinical infections, as this drug is used as last resort treatment for multiple antibiotic resistant enterococci (Ogier and Serror, 2008). Moreover, the transfer of vancomycin-resistance from *E. faecalis* to other pathogens such as methicillin-resistant *Staphylococcus aureus* has been reported in the last decade (Palmer et al., 2010). Virulence determinants also contribute to fitness and persistence of enterococci in nosocomial infections. Among the virulence factors that can be found in *E. faecalis*, some are sporadically detected in dairy isolates (Popović et al., 2018), like the aggregation substance (*asa1*), gelatinase (*gelE*), collagen adhesin (*ace*), enterococcal surface protein (*esp*) and cytolysin (*cylA*). For these reasons, *Enterococcus* species have not the GRAS status or recommendations for QPS list (EFSA Panel on Biological Hazards, Ricci et al., 2017), and the absence of transferable antibiotic resistance genes and/or potential virulence determinants should be investigated for each new potential probiotic strain intended to be proposed for food or pharmaceutical industry.

North African countries have an ancient tradition in food technology. Traditional dairy products and cheese remain very popular in Tunisia, consumed at breakfast, after lunch and at dinner. These traditional foods are still prepared at the household level and marketed through informal routes. The composition of these products is often not well characterized, varying with regions and local markets, sometimes using raw material of poor microbiological quality, or lacking hygiene conditions (Benkerroum, 2013). In order to improve the safety and quality of these foods, to promote the trading of such traditional foods internationally, and to be competitive in local markets, there is an urgent need for North African countries to develop and standardize new starters or probiotic strains in conformity with the Codex Alimentarius standards, WTO, FAO and EFSA legislations.

In this context, the aim of this work was to evaluate the probiotic potential and safety of *E. faecalis* OB14 and OB15, two strains isolated from traditional fermented Tunisian dairy products (Testouri cheese and Rigouta), in comparison to the known probiotic *E. faecalis* Symbioflor 1 DSM 16431.

## Materials and Methods

### Sampling and Isolation of Lactic Acid Bacteria (LAB)

A total of five samples of Tunisian traditional fermented dairy products (Testouri cheese, Rigouta, yogurt, Leben, Rayeb) were collected from local markets in different cities. 10 g or 10 mL of each dairy sample were suspended aseptically in 90 mL of sodium citrate solution (pH 7.0) and homogenized by vortexing. Each suspension was then serially diluted, in NaCl 0.9%, plated on Man-Rogosa-Sharpe (MRS) medium and incubated for 48 h at 37°C under anaerobic conditions to isolate Lactic Acid Bacteria (LAB). Single isolated colonies were picked and purified by repeated streaking, then grown overnight in MRS broth at 37°C and stored at -20°C in 20% (w/v) glycerol.

### Identification of OB14 and OB15

The isolates of LAB were presumptively identified by phenotypic analysis (colony morphology, Gram staining and catalase assay), and then by the simultaneous combination of the MALDI-TOF MS Biotyper system and species-specific PCR assay.

#### MALDI-TOF MS Biotyper

Bacteria were identified by analysis of the total proteome using an Autoflex III Matrix-Assisted Laser Desorption/ Ionization-Time-Of-Flight mass spectrometer (MALDI-TOF MS; Bruker, Marcy-l’Etoile, France) coupled to the MALDI-Biotyper 3.1 system, as previously described (Hillion et al., 2013; Zommiti et al., 2018). Formic acid was used on the bacterial spots as a quick extraction procedure (Haigh et al., 2011), then the MALDI target plate was introduced in the mass spectrometer for measurement and data acquisition. For each sample, 600 spectra were pooled, and the generated spectra were compared with the MALDI-Biotyper 3.1 database. A score was calculated based on the matching between the reference spectrum and the unknown spectrum. A score of ≥1.7 allows genus identification, and a score of ≥2.0 is the set threshold for identification at the species level (Sogawa et al., 2011). Dendrograms were generated from the minimal spanning tree data set (Elbehiry et al., 2017). For this, the main spectra of the Maldi Biotyper taxonomy were compared with the spectra resulting in a matrix of cross-wise identification scores. This matrix was applied to estimate the distance level of the bacteria for each pair of main spectra.

#### Species-Specific PCR Assay

Genomic DNA was extracted according to Delley et al. (1990). *E. faecalis* OB14 and OB15 were identified using the *E. faecalis* species-specific primers EflF1 (5′-ACCAATGTTGGCACAAGAAA-3′) and EflR1 (5′-TTTCGTTCAAG CGGTCTTTT-3′) as described by Park et al. (2017) with slight modifications. The PCR reactions were performed with the Thermo Scientific PCR Master Mix, in a total volume of 50 μL, using approximately 1 μM of forward and reverse primers and 10 pg–1 μg of DNA template. Amplification conditions were as follows: a first denaturation step at 94°C for 5 min, 30 cycles of denaturation at 94°C for 1 min, annealing at 56°C for 1 min, extension at 72°C for 1 min, followed by a final elongation step at 72°C for 10 min. The PCR products were analyzed on 1% agarose gel electrophoresis, stained with SYBR Safe DNA gel stain and examined under UV light.

*E. faecalis* OB14 and OB15 isolates, both correctly identified by MALDI-TOF MS Biotyper and species-specific PCR, were selected for the further experiments. Unless otherwise stated, the bacteria were routinely precultivated and cultivated in MRS broth at 37°C.

### Acid and Bile Salts Tolerance

For the acid tolerance test, the method described by Ramos et al. (2013) was utilized including some modifications. To simulate the acidic conditions of the gastrointestinal tract, MRS was adjusted to pH 3.0 with 1 N HCl (Azat et al., 2016). Bacterial cells from fresh overnight culture were centrifuged (10,000 ×*g*, 10 min), washed twice with Phosphate Buffer Saline (PBS), and resuspended to approximately 10^8^ bacteria/mL in the MRS broth pH 3.0. One mL of culture was taken immediately from each tube, diluted, and plated on MRS agar. Similarly, one mL was taken after 3 h of incubation at 37°C, reflecting the time spent in the human stomach. The colony forming units (CFU) were then counted, expressed as log 10 values of colony-forming units per milliliter (CFU/mL) and the bacterial survival rate (SR) was calculated.

The bile salts tolerance was tested as previously described by Anandharaj et al. (2015) with slight modifications. Freshly prepared overnight cultures were harvested by centrifugation and resuspended in MRS broth added with 0.3% bile salts (Oxgall, Sigma-Aldrich, France) followed by incubation at 37°C for 4 h, simulating the human intestine transit time. Aliquots were withdrawn at 0 and 4 h interval, diluted, and plated on MRS agar. Bile salts tolerance was assessed in terms of viable colony counts after aforesaid incubation at 37°C. The SR was calculated as described above for acid tolerance.

### Hydrophobicity and Autoaggregation

Hydrophobicity was estimated as previously reported by Rosenberg et al. (1980). Overnight cultures were centrifuged and resuspended at 10^8^ bacteria/mL. The cell suspensions (3 mL) were mixed with xylene (1 mL) for 2 min. After 1 h of incubation at room temperature, the aqueous phases were transferred to a spectrophotometry cuvette and the absorbance at 600 nm was measured. Hydrophobicity (%) was calculated as follows: H% = [(A_0_ - A)/A_0_] × 100, where A_0_ and A are absorbance values found before and after solvent extraction.

Autoaggregation test was performed as described by Del Re et al. (2000) and Collado et al. (2008) including slight modifications. Overnight cultures were centrifuged (10,000 ×*g*, 10 min), washed twice with PBS, and resuspended in this buffer to obtain a viable cell counts of approximately 10^8^ CFU/mL. Bacterial suspensions (4 mL) were vortexed for 15 s and incubated at room temperature. At time 0 and 24 h after incubation without mixing, 1 mL of the upper suspension was taken to measure the absorbance (A) at 600 nm. The autoaggregation was then calculated as follows: autoaggregation (%) = [1 - (A_24h_/A_0_) × 100].

### Biofilm Formation

Biofilm production on abiotic surfaces was studied in microtiter plates as performed by Bujnakova et al. (2014) and Gómez et al. (2016) with minor modifications. After 24 h incubation at 37°C, the adherent biofilms were gently washed, fixed, dried at room temperature, and stained with 0.1% crystal violet. Excess stain was rinsed off by placing the microtiter plates under running tap water. Then, the dye bound to adherent cells was removed with glacial acetic acid and the absorbance of the resulting solutions was measured at 595 nm. Biofilm formation was expressed using OD_C_ cut-off values as previously described (Extremina et al., 2011; Diaz et al., 2016), and the strains were then classified as belonging to one of the following categories: ODc < OD ≤ 2 × ODc = weak biofilm producer, 2 × ODc < OD ≤ 4 × ODc = moderate biofilm producer, and OD > 4 × ODc = strong biofilm producer.

### Gelatinase Assay

The gelatinase activity was investigated using Nutrient medium containing 3% (w/v) gelatin according to Ben Braïek et al. (2018). *E. faecalis* OG1RF was used as reference for quality control.

### Caco-2/TC7 Culture

Human enterocyte-like Caco-2/TC7 cell line was routinely grown in Dulbecco’s Modified Eagle’s Medium (DMEM, Invitrogen, France), supplemented with 15% heat-inactivated fetal bovine serum and Penicillin/Streptomycin, at 37°C in a humidified incubator with 5% CO_2_-95% air atmosphere. The medium was changed daily, and the cells were passaged and subcultured each week. For adhesion assay, cytotoxicity test and interleukine-10 (IL-10) quantification, the cells were seeded in 24-well tissue culture plates. For transepithelial electrical resistance (TER) measurement, the cells were grown on insert (3 μm pore size) for 21 days to ensure epithelial differentiation.

### Adhesion

Overnight bacterial cultures were harvested by centrifugation (10,000 ×*g*, 10 min), resuspended in cell culture medium without serum and antibiotic to achieve a concentration of 10^8^ bacteria/mL, and then applied on confluent Caco-2/TC7 monolayers. After 4 h of incubation at 37°C, in 5% CO_2_-95% air atmosphere, monolayers were washed with sterile pre-warmed PBS to remove non-adherent bacteria and lysed by incubation for 15 min with 0.1% Triton X-100. The lysates were then diluted and plated onto de Man, Rogosa and Sharpe (MRS) agar to determine the number of adherent bacteria.

### Hemolytic Activity and Cytotoxicity

Hemolytic activity was determined on Columbia agar containing 5% of sheep blood, by streaking the bacteria on the surface of the solid medium. The plates were then incubated at 37°C for 48 h under aerobiosis conditions and examined for β- (positive) or γ- (negative) hemolysis as previously indicated (Semedo et al., 2003).

The cytotoxicity of the *E. faecalis* strains was tested on Caco-2/TC7 cells following overnight incubation with the bacteria, using the Neutral Red (NR) uptake test and an enzymatic assay (Cytotox 96 Promega, France), as described by Messaoudi et al. (2012) and Biaggini et al. (2017), respectively.

### Quantification of the IL-10 Anti-inflammatory Cytokine

The level of interleukine 10 (IL-10) in the supernatants of Caco-2/TC7 cells was measured using the Human IL-10 ELISA Kit (Thermo Scientific), after overnight incubation of the cells with approximately 10^8^ bacteria/mL.

### Transepithelial Electrical Resistance

The effect of the bacteria on the TER of Caco-2/TC7 monolayers grown on inserts was measured using the Millicell Electrical Resistance system (Millipore, Bedford, MA, United States). The day before experiment, the inserts were washed with PBS to remove traces of antibiotics and the cell media was changed to the original media without antibiotics and serum. TER was measured before the addition of the bacterial suspension at 10^8^ bacteria/mL and during 16 h. TER values were expressed as percentages of the initial level measured in each insert.

### Antibiotic Susceptibility Testing

The antibiotic susceptibility of the *E. faecalis* strains was performed using the broth microdilutions method. The antibiotics selected were ampicillin, vancomycin, gentamicin and erythromycin. Overnight cultures of bacteria were inoculated at 10^5^ CFU/mL in Mueller-Hinton broth supplemented with various concentrations of the antibiotics (0.5, 1, 2, 4, 8, 16, 32, 64, 128, 256, and 516 μg/mL). The MIC for each antibiotic was confirmed as the lowest concentration at which no growth was observed following a 24 h incubation period at 37°C. The susceptibility/resistance were determined according to the microbial cut-off values recommended by the European Food Safety Authority [EFSA], 2012).

### Virulence Factors

Total DNA of *E. faecalis* OB14 and OB15, extracted previously for the Species-specific PCR assay, were used for PCR amplification of the following virulence determinants: aggregation substance (*asa1*), gelatinase (*gelE*), collagen adhesin (*ace*), enterococcal surface protein (*esp*) and cytolysin (c*ylA*). PCR experiments were performed using the primer sequences and protocols described in Mannu et al. (2003), Reviriego et al. (2005), and Anderson et al. (2016).

*Enterococcus faecalis* V583 and *E. faecalis* Symbioflor 1 DSM 16431 were used as positive and/or negative control. The PCR products were analyzed on 1% agarose gel electrophoresis, stained with SYBR Safe DNA gel stain and observed under UV light.

### Virulence in the *Galleria mellonella* Model

The *E. faecalis* strains used for infection of *G. mellonella* were grown for 24 h in M17 supplemented with 0.5% glucose (GM17). After centrifugation, bacterial cells were washed once in 0.9% NaCl and resuspended to a final OD_600__nm_ of 1 (5.10^7^ CFU/mL). The size of the inoculum was confirmed by numeration on solid GM17. Fifteen larvae were infected with 10 μL of a cell suspension through into the hemocoel using a microinjector (KDS100 Legacy, Fisher Scientific) with a sterilized microsyringe and incubated at 37°C. Larval surviving was monitored at 16 h, and then every hour until 24 h post-infection. *E. faecalis* V19 and *E. faecalis* Symbioflor 1 DSM 16431 were also tested under the same conditions as a virulent and non-virulent control, respectively.

### Sequencing and Analysis of Whole Genome

Genomic DNA quality and quantity of *E. faecalis* OB14 and OB15 were examined on a 1% agarose gel electrophoresis, also using a NanoDrop^TM^ spectrophotometer and a Qubit^®^^TM^ 4.0 fluorometer (Qubit^®^ dsDNA HS assay). DNA libraries for whole genome sequencing (WGS) were constructed using Nextera^TM^ XT DNA Library Prep Kit according to the manufacturer’s instructions (Illumina, San Diego, CA, United States). The sequencing was performed on the Illumina MiSeq platform (LMSM Evreux, Rouen Normandy University) using Reagent Kit v3 (600-cycle, Illumina, United States) to generate 2 × 250 bp paired-end reads.

Paired-end reads were trimmed using Trimmomatic v.0.36 (Bolger et al., 2014). Sequence data quality was checked using FastQC v.0.11.6.^1^ Assembly of paired-end reads was done *de novo* using Spades software package v.3.12.0 (Bankevich et al., 2012) using its in-build read error correction functionality of BayesHammer (Nikolenko et al., 2013) by setting the “carefull” option, and *k*-mer combination was set to 21, 33, 55, 77, 99 and 127. The obtained drafts were checked for consistency, such as the number of contigs, N50, GC% and total size of assembly using Quast v.5.0.0 (Mikheenko et al., 2018).

In addition to MALDI-TOF and specific PCR assays, confirmation of cluster analysis was achieved using WGS data with two methods: (i) average nucleotide identity (ANI) was calculated using PYANI v0.2.7 (Pritchard et al., 2016; Pritchard, 2017), and (ii) genome-to-genome distance calculator (GGDC), which is an *in silico* DNA-DNA hybridization method (*is*DDH) using a webserver at ggdc.dsmz.de/ggdc.php (Meier-Kolthoff et al., 2013). The *is*DDH model “formula 2” was used as recommended for draft genomes.

MLST (Multilocus sequence typing) profile of each isolate was determined from the draft genome sequences using the software package MLST_check v. 2.1.1706216-1 (Page et al., 2016)^2^ based on the *E. faecalis* PubMLST database^3^.

ABRicate v.0.8.7 (Seemann, 2018), a mass screening software package of contigs, for resistome and virulome coding-genes detection, was used to screen against the NCBI Bacterial Antimicrobial Resistance Reference Gene Database (NCBI BARRGD, PRJNA313047), and Virulence Factors of Pathogenic Bacteria Database (VFDB; Chen et al., 2016), respectively.

### Statistical Analysis

Results are expressed as means ± standard error (SE) of three experiments done in triplicate. Analysis of statistical significance was performed with Student’s *t*-test and GraphPad Prism7.

## Results and Discussion

### Identification of the LAB Isolates OB14 and OB15

Lactic acid bacteria strains were isolated from traditional fermented Tunisian dairy products (Testouri cheese, Rigouta, yogurt, Leben, Rayeb). Two *Enterococcus* strains (Gram positive, catalase negative, coccus morphology), OB14 and OB15, respectively found in Testouri cheese and Rigouta from two different Tunisian regions, were selected for MALDI biotyper and species-specific PCR assays, for confirmation of the genus *Enterococcus* and determination of the species. The analysis by MALDI biotyper classified OB14 and OB15 isolates as *E. faecalis* strains with score value >2.0, which allows to identify at the species level with no doubt (Sogawa et al., 2011).

PCR amplification with *E. faecalis* specific primers (Park et al., 2017) confirmed results found by MALDI biotyper. As expected, a PCR product of 1,209 bp was obtained for the two isolates and for the probiotic *E. faecalis* Symbioflor 1 DSM 16431, corresponding to the specific amplification of the 6-aminohexanoate-cyclic-dimer hydrolase (EC 3.5.2.12) target gene of *E. faecalis.*

### Hierarchical Cluster Analysis

**FIGURE 1:**
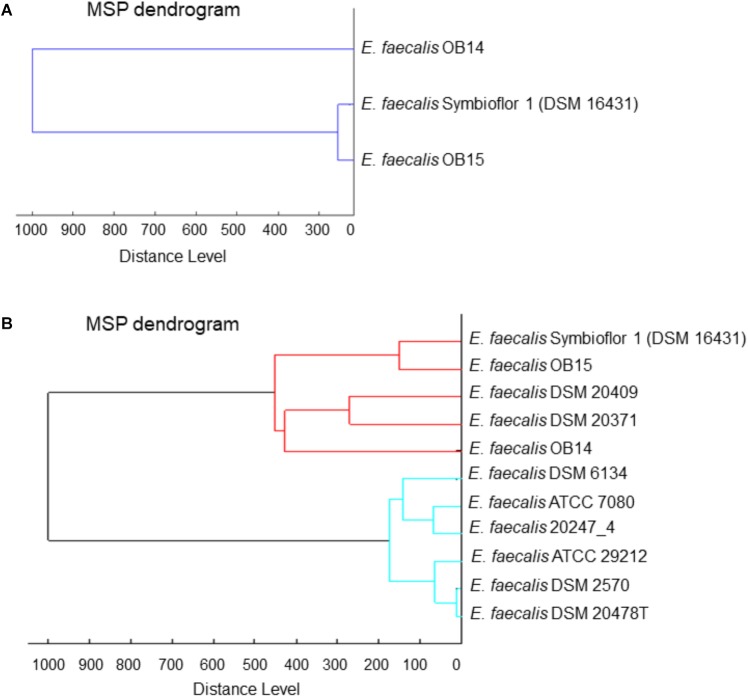
Dendrograms (representations of hierarchical cluster analysis) of the Tunisian dairy isolates *Enterococcus faecalis* OB14 and *E. faecalis* OB15,**(A)** compared to *E. faecalis* Symbioflor 1 DSM 16431 and **(B)** to the reference strains of the Biotyper library.

Besides bacterial identification, MALDI-TOF MS allows hierarchical cluster analysis, using an algorithm to determine the relatedness between the MS spectrum of each bacteria. MS spectra of *E. faecalis* OB14 and OB15 were compared to the MS spectrum of the reference probiotic strain *E. faecalis* Symbioflor 1 DSM 16431, and to MS spectra of the *E. faecalis* strains available in the Biotyper library. For this, spectra were merged, and the merging patterns were then represented as dendrograms or tree structures (Figure 1A,B). Since spectra were merged by relatedness, the distance of branches on the dendrograms relates directly to the similarity of spectra and, hence, the similarity of the bacteria studied. Unexpectedly, *E. faecalis* OB15 was found to be closely related to Symbioflor 1 DSM 16431 than to OB14, the other Tunisian dairy isolate (Figure 1A), or to the reference strains of the Biotyper library (Figure 1B).

**FIGURE 2:**
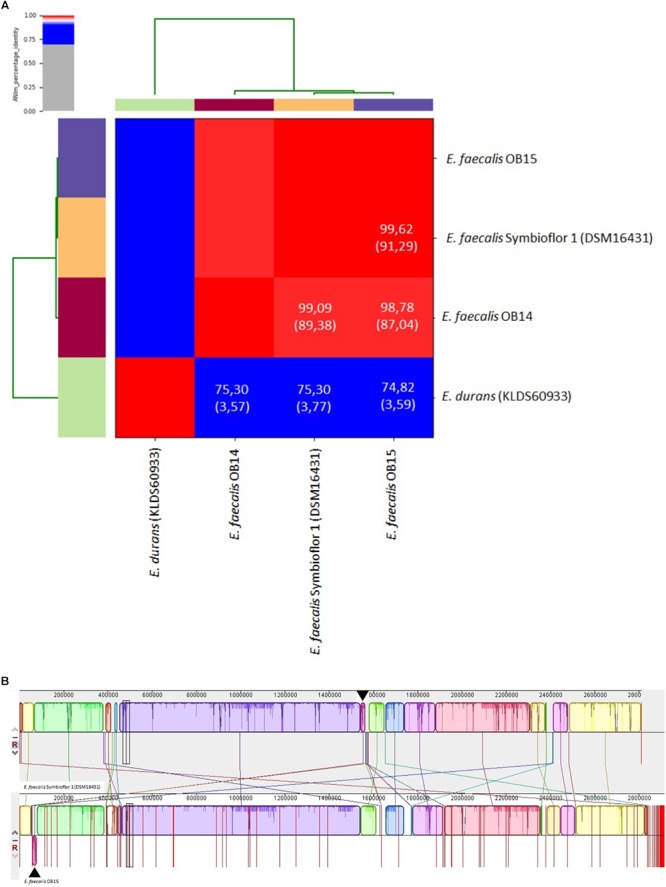
**(A)** Heat-map of Average Nucleotide Identity (ANI) between *Enterococcus faecalis* OB14, *E. faecalis* OB15 and *E. faecalis* Symbioflor 1 DSM 16431. ANI values (%) and coverage (between brackets) are also indicated. **(B)** Pairwise alignment between the reference strain Symbioflor 1 and the related strain OB15 using the MAUVE software. Colored blocks outline genome sequences that align to the other genome and are presumably homologous and internally free of genomic rearrangement (Locally Colinear Blocks or LCBs). White regions correspond to sequences that are not aligned and probably contain sequence elements specific to a genome. Blocks below the center line indicate regions that aligned in the reverse complement (inverse) orientation. The height of the profile within each LCB demonstrates the average degree of sequence conservation within an aligned region.

Whole genome analysis using the ANI and the Genome-to-Genome Hybridization similarity methods also showed that *E. faecalis* OB15 is closer to Symbioflor 1 DSM 16431 than to OB14 in terms of genomic distance (Figure 2A). This agrees with hierarchical cluster analysis from MALDI-TOF MS.

To complete these data, the overall genomic differences between *E. faecalis* Symbioflor 1 DSM 16431 and the OB15 strain have been investigated by aligning the two genomes using MAUVE v.20150226 (Figure 2B; Darling et al., 2004). This alignment suggests a high level of conservation along the chromosome. Indeed, OB15 and Symbioflor 1 DSM 16431 genomes were found to be organized in a very similar way, except one inversion of physically close syntenic block highlighted by arrows.

MLST analysis allowed to identify sequence types (ST) of *E. faecalis* OB15 and *E. faecalis* Symbioflor 1 DSM 16431, by comparing their sequences with alleles from the *E. faecalis* MLST database. This showed that the MLST profile of OB15 and Symbioflor 1 DSM 16431 are not similar (OB15 belongs to clone ST25 and Symbioflor 1 DSM 16431 is clone ST248).

Once identified, *E. faecalis* OB14 and OB15 were then evaluated for their probiotic potential using physiological tests and *in vitro* analysis (gastric and intestinal transit tolerance, hydrophobicity and autoaggregation, biofilm/adhesion on abiotic surface, adhesion to intestinal cells, production of the anti-inflammatory cytokine IL-10, modulation of the epithelial barrier).

### Probiotic Potential of OB14 and OB15

#### Gastric and Intestinal Transit Tolerance

An effective property as prerequisite for probiotics is their ability to resist to the harsh condition in the stomach and the small intestine (Ouwehand et al., 2002). Therefore, *E. faecalis* OB14 and OB15 were tested for their ability to survive in acidic condition and in the presence of 0.3% bile salts, compared to the *E. faecalis* Symbioflor 1 probiotic strain. Table 1 showed that after 3 h of incubation at pH 3.0, *E. faecalis* OB14 and OB15 retained their viability with 84.7 and 97.3% survival rate, respectively. *E. faecalis* OB15 showed a highly similar viability to Symbioflor 1 (97.4%), whereas the survival of *E. faecalis* OB14 was slightly affected (-15%).

**Table 1:** Acid and bile salts tolerance of *Enterococcus faecalis* OB14, *E. faecalis* OB15 and *E. faecalis* Symbioflor 1 DSM 16431.

	Acid tolerance			Bile salts tolerance		
Strains	Viable counts (log CFU/mL)		Survival rate (%)	Viable counts (log CFU/mL)		Survival rate (%)
	T0	T3		T0	T4	
*E. faecalis* OB14	7.8 ± 0.2	6.6 ± 0.1	84.7	8.2 ± 0.3	6.9 ± 0.2	85.2
*E. faecalis* OB15	7.4 ± 0.4	7.2 ± 0.2	97.3	7.8 ± 0.1	6.7 ± 0.3	86.1
*E. faecalis* Symbioflor 1 (DSM 16431)	7.8 ± 0.3	7.6 ± 0.2	97.4	7.9 ± 0.3	6.8 ± 0.4	87.0


*Values are mean of triplicate ± standard error*.

A genome analysis of *E. faecalis* Symbioflor 1, performed by Domann et al. (2007) revealed the presence of genes that mediate resistance against oxidative stress, and may facilitate the bacteria to survive exposure to gastric acid following consumption, and to survive and proliferate in the intestine. Haghshenas et al. (2016) have also reported that *Enterococcus* strains showed better low pH tolerance than many LAB strains belonging to *Lactobacillus*, *Lactococcus* or *Leuconostoc* genus. This high tolerance capability can be associated to the bilayer membrane structure, which allows easy tolerance of inverse conditions.

In the current study, the tolerance of *E. faecalis* OB14 and OB15 to 0.3% bile salts was also tested (Table 1). This concentration has been recommended as suitable for screening probiotic, as it simulates the conditions within the gastrointestinal tract (Goldin and Gorbach, 1992). The two isolates displayed high tolerance to bile salts, after 4 h of incubation, with a survival >80%, similarly to *E. faecalis* Symbioflor 1 DSM 16431, the probiotic reference added in the experiment (Table 1). These findings are in accordance with previous studies that showed that *Enterococcus* species had the highest tolerance capability in the bile salts conditions among LAB group (Klaenhammer and Kullen, 1999). For example, it has been found that *E. faecalis* 13C can strongly tolerate 0.3% oxgall with a survival rate of 98% after 3 h of incubation (Haghshenas et al., 2016), and *E. faecalis* CP58 was capable to retain viability for 6 h with a survival rate of 92.6% (Nueno-Palop and Narbad, 2011). Some strains of *Enterococci*, isolated from canine feed, were even found to be able to grow in the presence of 5% Oxgall (Lauková et al., 2008).

#### Hydrophobicity and Autoaggregation

**FIGURE 3:**
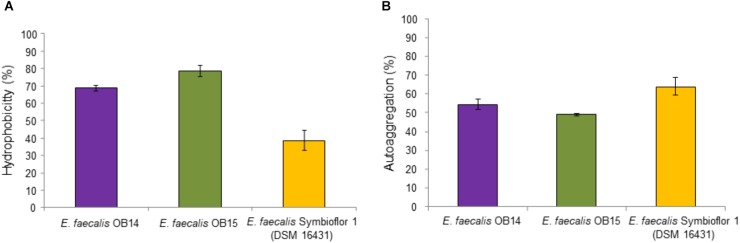
Hydrophobicity **(A)** and autoaggregation **(B)** of *Enterococcus faecalis* OB14, *E. faecalis* OB15 and *E. faecalis* Symbioflor 1 DSM 16431.

Hydrophobicity is a physicochemical feature related to the capacity of bacteria to autoaggregate and adhere to various types of surfaces including eukaryotic cells. Thus, the affinity of *E. faecalis* OB14 and OB15, and Symbioflor 1 DSM 16431 toward the hydrophobic solvent xylen was examined. The results of this experiment showed 68.8, 78.6, and 38.6% hydrophobicity, respectively (Figure 3A). Concerning autoaggregation (Figure 3B), the probiotic strain *E. faecalis* Symbioflor 1 DSM 16431, used as reference, showed the higher percentage reaching 63.9%, whereas the scores for *E. faecalis* OB14 and OB15 strains were 54.3 and 48.9%, respectively. These percentages are consistent to the autoaggregation of *E. faecalis* 14 (49%), that has been isolated from the meconium of human donors and considered as high autoaggregative strain (Al Atya et al., 2015). Hydrophobicity and autoaggregation are beneficial attributes for probiotics, necessary for adhesion to intestinal cells, and to form a barrier that prevents the colonization by food-borne pathogens (Pelletier et al., 1997; Dunne et al., 2001; Collado et al., 2008).

#### Biofilm/Adhesion on Abiotic Surface

Biofilm-forming abilities of *E. faecalis* OB14 and OB15, and *E. faecalis* Symbioflor 1 DSM 16431, were evaluated on abiotic surfaces. The three strains were found to be weak to moderate biofilm producers (data not shown). Adhesion to abiotic surfaces is not routinely investigated in probiotic strains, even if a positive correlation often exists between biofilm formation on abiotic surfaces and adhesiveness to biotic surfaces (Bujnakova et al., 2014). Thus, to better estimate adhesion of the isolates to the gut, this parameter was then investigated on Caco-2/TC7 intestinal cells.

#### Adhesion to Intestinal Cells

Bacterial adhesion to epithelial cells is considered as one of the most reliable features for selection criteria of probiotic strains (Garcia-Gonzalez et al., 2018). Caco-2 cell line has been frequently used as an *in vitro* model to screen for adhesive strains (Elo et al., 1991; Chauvière et al., 1992; Bernet et al., 1994). In this study, the adhesion of *E. faecalis* OB14 and OB15 to Caco-2/TC7 cells was investigated and compared to the adhesion of *E. faecalis* Symbioflor 1 DSM 16431. The results found showed adhesive potential in the range of 3–12% for the three strains. This agrees with other reports that have investigated the capacity of attachment of *E. faecalis* to cell lines. For example, a rate of approximately 2% adhesion was recently found for *E. faecalis* B3A-B3B, a bacteriocinogenic strain that has been isolated from a healthy Iraqi infant (Al Seraih et al., 2018). Indeed, after treatment of the Caco-2 cells with 10^7^ bacteria for 2 h, the authors recovered 1.8 10^5^ CFU/mL of adherent bacteria. Similarly, Nueno-Palop and Narbad (2011) have previously found an adhesion rate of 2.6% for *E. faecalis* CP58 isolated from human gut. These values have been considered has high capability to adhere to Caco-2 cells for *Enterococcus* strains with dairy origin (Cebrián et al., 2012; Pimentel et al., 2012).

It has been reported that the mechanism of adhesion to Caco-2/TC7 cells involves the combinations of carbohydrate and protein factors on the bacterial cell surface (Kimoto-Nira et al., 2007). In *Enterococcus*, the aggregation substance, is known to play a role in adherence to eukaryotic cells (Mundy et al., 2000). Aggregation factors may be advantageous for probiotic strains, since it can help the bacterium, to colonize the intestine after consumption, to proliferate and hence to display its probiotic traits (Veljović et al., 2017).

#### Production of IL-10

**FIGURE 4:**
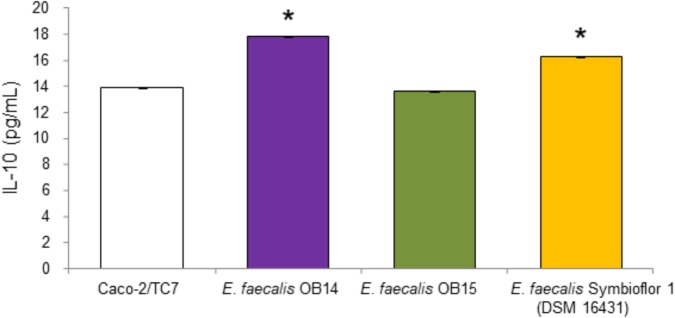
Quantification of IL-10 in the supernatants of Caco-2/TC7 cells after treatment with *Enterococcus faecalis* OB14, *E. faecalis* OB15 and *E. faecalis* Symbioflor 1 DSM 16431. ^∗^*P* < 0.05 compared to Caco-2/TC7.

The ability of LAB to modulate cytokines production is considered as another criterion for the selection of beneficial microbes (Amit-Romach et al., 2010). IL-10 has interesting impact on immunoregulation, since it inhibits type 1/proinflammatory cytokine formation (Asadullah et al., 1998). Therefore, we quantified the IL-10 secretion by Caco-2/TC7 cells to investigate the ability of *E. faecalis* OB14 and OB15 to modulate inflammatory reaction in human intestinal cell lines, compared to the effect of *E. faecalis* Symbioflor 1. The results obtained by the ELISA assay (Figure 4), showed that *E. faecalis* OB14 induced a 28% increase of IL-10 secretion by Caco-2/TC7 cells (production of 17.8 pg/mL compared to 13.9 pg/mL for the basal cytokine value); *E. faecalis* Symbioflor 1 induced a 17% increase (production of 16.3 pg/mL), whereas *E. faecalis* OB15 had no effect. These differences among bacteria are not surprising since the immunomodulatory effects are often strain-specific (Helwig et al., 2006; Snel et al., 2011). Indeed, Al Seraih et al. (2018) have studied the anti-inflammatory effect of the B3A-B3B *E. faecalis* strain, isolated from an infant faces, and found that this bacterium had no effect on IL-10 production by Caco-2 cells. Conversely, Are et al. (2008) demonstrated that *E. faecalis* EC16, isolated from a healthy newborn baby, could induce the anti-inflammatory cytokine IL-10 in the intestine through PPAR-gamma, and eliminate the inflammatory responses. Similarly, Wang et al. (2014) showed that *E. faecalis* from healthy infants modulates inflammation through MAPK signaling pathways.

#### Modulation of the Epithelial Barrier

**FIGURE 5:**
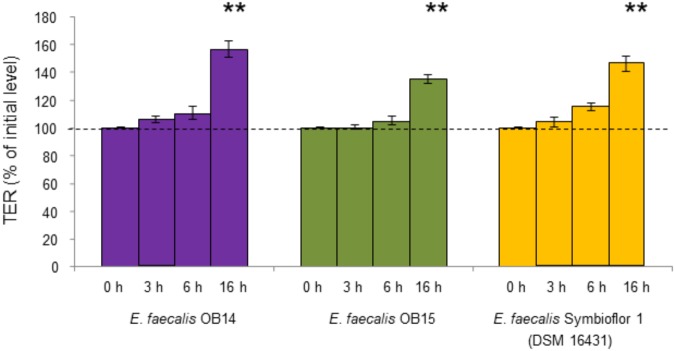
TER of Caco-2/TC7 cells exposed to *Enterococcus faecalis* OB14, *E. faecalis* OB15, and *E. faecalis* Symbioflor 1 DSM 16431. ^∗∗^*P* < 0.01 compared to initial value.

Changes in the TER of polarized Caco-2/TC7 cells were used as an indicator of the effect of *E. faecalis* OB14 and OB15 on the intestinal epithelial barrier function (Figure 5). The results showed that the bacteria maintained the TER almost constant for the first 6 h compared to the initial value in the insert. After 16 h, *E. faecalis* OB14 and OB15, increased the TER, similarly to *E. faecalis* Symbioflor 1. Lodemann et al. (2015) also showed that a strain of *Enterococcus* (*E. faecium* NCIMB 10415) can enhance the TER when administered at a concentration of 10^6^/1.12 cm^2^.

After the investigation of these beneficial attributes for *E. faecalis* OB14 and OB15 as probiotic candidates, we examined the safety of the bacteria using various tests (antibiotic susceptibility, hemolysis and cytotoxicity, virulence).

### Safety Evaluation of OB14 and OB15

#### Antibiotic Susceptibility

One of the required properties by which specific strains can be considered as a potential probiotic bacterium is that they must not harbor acquired and transferable antibiotic resistances (Courvalin, 2006; Zheng et al., 2017). Table 2 shows the MICs of *E. faecalis* OB14 and OB15 to the four selected antibiotics from two different groups: cell wall inhibitors (ampicillin and vancomycin) and protein synthesis inhibitors (gentamicin and erythromycin), compared to the *E. faecalis* Symbioflor 1 reference strain. Strains were considered resistant when they showed MIC values higher than the MIC breakpoints established by the EFSA (2012). The three strains exhibited the same pattern of sensitivity/resistance to the antibiotic tested. They were sensitive to the clinically important antibiotics ampicillin (MIC = 1 μg/mL) and vancomycin (MIC = 2 μg/mL), and resistant to gentamicin (MIC = 128 μg/mL and erythromycin (MIC > 256 μg/mL).

**Table 2:** Antibiotic susceptibility of *Enterococcus faecalis* OB14, *E. faecalis* OB15 and *E. faecalis* Symbioflor 1 DSM 16431.

	Minimum inhibitory concentrations (MICs in μg/mL)			
	Ampicillin	Vancomycin	Gentamicin	Erythromycin
*E. faecalis* OB14	1 (S)	2 (S)	128 (R)	>256 (R)
*E. faecalis* OB15	1 (S)	2 (S)	128 (R)	>256 (R)
*E. faecalis* Symbioflor 1 DSM 16431	1 (S)	2 (S)	128 (R)	>256 (R)


*(S) and (R): Susceptible and Resistant strain, according to the breakpoints established by EFSA. The EFSA Enterococcal breakpoints (mg/L): 2 mg/L (ampicillin), 4 mg/L (vancomycin), 32 mg/L (gentamicin) and 4 mg/L (erythromycin)*.

Genomic analysis was used to complete these data and showed absence of genes conferring resistance to vancomycin, in both *E. faecalis* OB14 and OB15. Furthermore, sequencing data showed that *E. faecalis* OB14 harbored tetracycline resistance determinants unlike to *E. faecalis* OB15 and *E. faecalis* Symbioflor 1.

Various opinions exist as to whether it might be desirable that some probiotic strains show resistance to specific antibiotics. On the one hand, some lactic acid bacteria can serve as host for antibiotic resistance genes and transfer these genes to pathogens. However, many of the resistance observed are intrinsic or natural resistance and non-transmissible (Salminen et al., 1998; Voravuthikunchai et al., 2006). It gives advantages in the clinical application, as this allows the probiotic to be taken at the same time as the antibiotic treatment. Resistance of enterococci to gentamicin has been described as intrinsic and partially attributed to a poor uptake of the antibiotic (Kristich et al., 2014). Resistance to erythromycin seems to be intrinsic as well, non-transmissible, and is widely spread among enterococci commonly found in foods (Barbosa et al., 2009). Recently, a high rate of erythromycin resistance has also been detected among Enterococci isolated from Artisanal Tunisian Meat “Dried Ossban” (Zommiti et al., 2018). Macrolides are frequently used in animal husbandry; this could contribute to the emergence of many resistant strains (Diarra et al., 2010). The most frequent type of macrolide resistance is the production of a methylase enzyme, encoded by erm genes (MLSB phenotype) which specifically methylates an adenine residue in the 23S rRNA of the 50S ribosomal subunit. This reduces the binding affinity of macrolides for the ribosome and hence renders macrolides ineffective (Kristich et al., 2014). Another mechanism of resistance is due to the presence of an efflux pump system mediated by the membrane-bound efflux protein, encoded by mef (A/E) and msr genes. Finally, in a study conducted by Anderson et al. (2016), tetracycline-resistance was also found to be frequently present in food (13/14 isolates tested). Besides the presence of some antibiotic’s resistance in *Enterococcus* strains in foods, a recent work concludes that *E. faecalis* isolated from raw milk cheeses does not represent a substantial reservoir of antimicrobial resistance and virulence factors if compared with clinical strains (Silvetti et al., 2019).

#### Hemolysis and Cytotoxicity

To develop or select new beneficial microbes, their absence of hemolysis capacity and/or cytotoxicity should be assessed at first (Salminen et al., 1998). Indeed, although some strains of *Enterococcus* have been previously used for their technological properties, or as probiotic for humans and animals, a case-by-case-evaluation is needed (Araújo and Ferreira, 2013; Hanchi et al., 2018).

**FIGURE 6:**
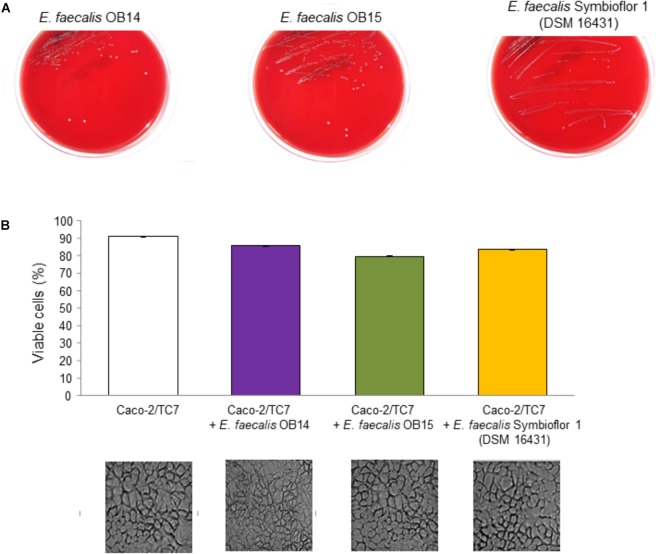
Hemolysis assay **(A)** of *Enterococcus faecalis* OB14, *E. faecalis* OB15 and *E. faecalis* Symbioflor 1 DSM 16431 on Columbia agar containing 5% of sheep blood. Viability of Caco-2/TC7 cells **(B)** measured using the Neutral Red uptake assay and observed by microscopy after treatment with 10^8^ bacteria/mL of *E. faecalis* OB14, *E. faecalis* OB15 and *E. faecalis* Symbioflor 1 DSM 16431, compared to untreated Caco-2/TC7 cells.

As mentioned above, hemolytic activity has been determined on Columbia agar containing 5% of sheep blood, by streaking the bacteria on the surface of the solid medium. The results obtained after 48 h incubation at 37°C under aerobic conditions are presented on Figure 6A. Observation of the Columbia agar plates showed no hemolysis zones of red blood cells (γ-hemolysis) for *E. faecalis* OB14 and OB15 similarly as the *E. faecalis* Symbioflor 1 probiotic strain. However, erythrocytes from different species were previously found to show various levels of susceptibility to hemolysin-mediated lysis. Sheep red blood cells were less susceptible than rabbit, human and horse red blood cells (Semedo et al., 2003). Thus, to complete this analysis, as no hemolysis has been observed in the present work, the cytotoxicity of *E. faecalis* OB14 and OB15 was also studied on the Caco-2/TC7 intestinal cell line. The NR uptake assay, used to quantify the viable cells, showed high rates of eukaryotic cell survival ranging from 79.8 to 85.9%, after contact with the three bacteria (Figure 6B). In accordance with these results, the cytotoxicity test using Cytotox 96 kit displayed mortality below 10% after incubation with the bacteria, similarly to natural death of cells (data not shown).

The absence of cytotoxicity of *E. faecalis* OB14 or OB15, compared to *E. faecalis* Symbioflor 1 DSM 16431, was reinforced by microscopic observations which did not show any damage of the cell monolayers (Figure 6B). These results agree with the known safety of *E. faecalis* Symbioflor 1 (Christoffersen et al., 2012; Fritzenwanker et al., 2013). This bacterium has been shown to not be toxic in an *in vivo Caenorhabiditis elegans* model and has been used as probiotic for humans for many years without triggering infection (Domann et al., 2007; Neuhaus et al., 2017).

### Analysis of Virulence Genes

The investigation of virulence determinants is an absolute prerequisite to characterize *E. faecalis* strains and classify them as probiotics or conversely potential pathogens, and to avoid the risk of dissemination of virulence factors to other bacteria by genetic transfer. Thus, presence/absence of the aggregation substance (*asa1*), gelatinase (*gelE*), collagen adhesin (*ace*), enterococcal surface protein (*esp*) and cytolysin (c*ylA*) genes were checked by PCR assays and whole genome sequencing (WGS; Table 3). The *ace* and *esp* genes were not found in *E. faecalis* OB14 and OB15 whereas *asa1* and *gelE* gene were detected by both methods. These results are not surprising as Domann et al. (2007) showed that the genotype of the *E. faecalis* Symbioflor 1 probiotic was also *esp*^-^ and *agg* (*asa*1)^+^, but conversely for the two other genes, these authors found that the strain was *ace*+ and *gelE*^-^. However, despite the presence of *gelE* gene in *E. faecalis* OB14 and OB15, these bacteria failed to degrade gelatin on Nutrient gelatin medium (data not shown). This gene may be silent or expressed at a low level in our experimental conditions. Besides, all the genes belonging to the cytolysin operon (*cylA, cylB, cylI, cylL, cylM*, and *cylS*) were found in *E. faecalis* OB14, but not in OB15. Due to the detection of some virulence genes in *E. faecalis* OB14 and OB15, a test of safety/virulence was then performed on the *Galleria mellonella in vivo* model.

**Table 3:** Potential virulence factors of *Enterococcus faecalis* OB14 and *E. faecalis* OB15 detected by PCR and genomic analysis.

Gene	Product	*E. faecalis* OB14		*E. faecalis* OB15	
		PCR	Genomic analysis	PCR	Genomic analysis
*asa1*	Aggregation substance	+	+	+	+
*gelE*	Gelatinase	+	+	+	+
*Ace*	Collagen adhesion protein	-	-	-	-
*Esp*	Enterococcal surface protein	-	-	-	-
*cylA*	Cytolysin activator	+	+	-	-
*cylB*	Cytolysin transport	nd	+	nd	-
*cyllI*	Cytolysin immunity	nd	+	nd	-
*cyllL*	Cytolysin precursor	nd	+	nd	-
*cyllM*	Cytolysin modification	nd	+	nd	-
*cylS*	Cytolysin precursor	nd	+	nd	-


*nd, not determined*.

### Virulence Test on *Galleria mellonella* Model

**FIGURE 7:**
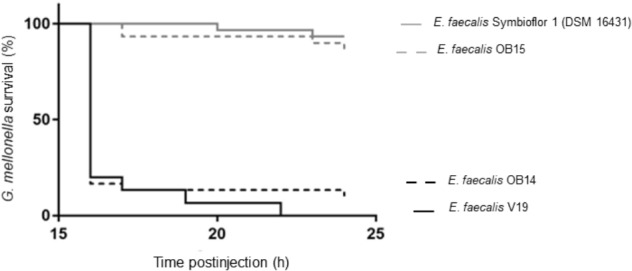
*Galleria mellonella* survival following injection with *Enterococcus faecalis* OB14, *E. faecalis* OB15, *E. faecalis* Symbioflor 1 DSM 16431 or *E. faecalis* V19.

Mammalian models of infection are costly and may raise ethical issues. As a reliable alternative, larvae of *Galleria mellonella* have been shown to be a powerful infection model to investigate virulence of various pathogens including *E. faecalis* (Ramarao et al., 2012). Thus, the virulence of *E. faecalis* OB14 and OB15 was estimated using this infection model, compared to *E. faecalis* Symbioflor 1 DSM 16431 (probiotic strain) and *E. faecalis* V19 (pathogenic strain). Larval surviving was monitored at 16 h, and then every hour until 24 h post-injection (Figure 7). The results show that *G. mellonella* larvae were able to survive inoculation of *E. faecalis* OB15 with the same survival profile that after injection with *E. faecalis* Symbioflor 1 DSM 16431, so this strain isolated from Rigouta can be considered as non-virulent and might be interesting for further development as potential probiotic. On the contrary, despite its food origin, *E. faecalis* OB14, isolated from Testouri cheese, led to a fast and drastic mortality of the larvae by 16 h post-injection, which was quite as toxic as the *E. faecalis* V19 lethal positive control of pathogenic strain. These results are in concordance with the presence of the cytolysin genes found in the genome of *E. faecalis* OB14, as cytolysin has been previously linked to virulence enhancement in animal models infected by *E. faecalis* and acute patient mortality in the clinic (Tang et al., 2018). By contrast, cytolysin genes were absent in *E. faecalis* OB15 and the bacterium was not toxic for the larvae. Taking together, all these results showed the necessity to follow the recommendations of guidance for selection of *Enterococcus* strains as probiotic, using intensive case by case evaluation for each strain, physiological analysis, whole genome sequencing and virulence models, even if the strains are isolated from a food ecological niche. Indeed, the risk of contamination with a pathogen strain during traditional process, due to a lack of hygiene or a poor quality of the raw material that has been used cannot be ruled out.

## Conclusion

*In vitro* testing is a rational starting point for evaluating the safety of bacteria, to select potential probiotic or to establish their possible mechanisms of action, since *in vivo* methods are costly and need complex agreement by ethical committees. In the current study, it has been found that *E. faecalis* OB14 and OB15 can adhere to intestinal cells, tolerate gastric and intestinal conditions (acidity, bile), and demonstrate the capacity to reinforce the epithelial barrier, similarly to the known probiotic reference strain, *E. faecalis* Symbioflor 1 DSM 16431. However, despite its food origin, *E. faecalis* OB14 was found to harbor resistance to tetracycline and some important virulence traits as the presence of cytolysin genes. This has been confirmed by the *in vivo* test on *Galleria Mellonella* which led to a high percentage of larvae mortality, so this bacterium can not be selected for probiotic candidate. On the contrary, *E. faecalis* OB15, isolated from Rigouta, seems to be an interesting strain for further studies and probiotic development for traditional fermented food and feed industry.

## Author Contributions

MM, MF, FA, and NC designed the study. OB and NC carried out most of the experiments, analyzed the data, and drafted the manuscript. LF participated in the sampling and isolation of bacteria. KZ and ED provided the probiotic reference strain. AZ, MC, and OM contributed to the PCR assays. MB helped with the identification by MALDI-TOF MS. AB and OM conducted whole genome sequencing experiments and bioinformatic analysis. IR and CM performed the virulence test on the *Galleria mellonella* model. FA and NC co-supervised the entire project. All authors read and approved the final manuscript.

## Conflict of Interest Statement

KZ was employed by company SymbioPharm GmbH and provided the *E. faecalis* Symbioflor 1 strain as probiotic reference for the experiments. The remaining authors declare that the research was conducted in the absence of any commercial or financial relationships that could be construed as a potential conflict of interest.

## Footnotes

1 http://www.bioinformatics.babraham.ac.uk/projects/fastqc

2 https://github.com/sanger-pathogens/mlst_check

3 https://pubmlst.org/efaecalis/ (accessed February 19, 2019).
